# Real-Time Immunosensor for Small-Molecule Monitoring in Industrial Food Processes

**DOI:** 10.1021/acs.analchem.3c00628

**Published:** 2023-05-13

**Authors:** Chris Vu, Yu-Ting Lin, Stijn R. R. Haenen, Julia Marschall, Annemarie Hummel, Simone F. A. Wouters, Jos M. H. Raats, Arthur M. de Jong, Junhong Yan, Menno W. J. Prins

**Affiliations:** † Department of Biomedical Engineering, Eindhoven University of Technology, 5612 AZ Eindhoven, The Netherlands; ‡ Institute for Complex Molecular Systems (ICMS), Eindhoven University of Technology, 5612 AZ Eindhoven, The Netherlands; § Helia Biomonitoring, 5612 AR Eindhoven, The Netherlands; ∥ AbSano, 5349 AB Oss, The Netherlands; ⊥ Department of Applied Physics and Science Education, Eindhoven University of Technology, 5612 AZ Eindhoven, The Netherlands

## Abstract

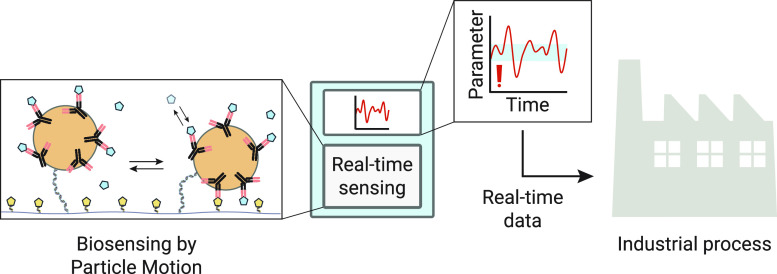

Industrial food processes are monitored to ensure that food is being produced with good quality, yield, and productivity. For developing innovative real-time monitoring and control strategies, real-time sensors are needed that can continuously report chemical and biochemical data of the manufacturing process. Here, we describe a generalizable methodology to develop affinity-based biosensors for the continuous monitoring of small molecules in industrial food processes. Phage-display antibody fragments were developed for the measurement of small molecules, as exemplified with the measurement of glycoalkaloids (GAs) in potato fruit juice. The recombinant antibodies were selected for use in a competition-based biosensor with single-molecule resolution, called biosensing by particle motion, using assay architectures with free particles as well as tethered particles. The resulting sensor measures GAs in the micromolar range, is reversible, has a measurement response time below 5 min, and enables continuous monitoring of GAs in protein-rich solutions for more than 20 h with concentration measurement errors below 15%. The demonstrated biosensor gives the perspective to enable a variety of monitoring and control strategies based on continuous measurement of small molecules in industrial food processes.

## Introduction

Industrial food manufacturing plants use series of processing steps to make products that are tasty and nutritious. A central challenge in the manufacturing of food and food ingredients is to deal with variations that are always present in the input materials and in different processing steps. These variations make it difficult to produce the final products with constant quality and to achieve optimal yield and productivity of the manufacturing processes.

A sector of industrial food processing with strong innovations is the field of plant-based proteins due to societal interest in food that is produced in resource-efficient and environmentally sustainable ways without using animals.^1,2^ Processes are being developed to extract and purify proteins from sources such as grains, pulses, and potatoes. These processes include the removal of so-called anti-nutritional factors, which are substances that reduce the nutritional value of the food product. An example are the glycoalkaloids (GAs), a family of small-molecule compounds that occur naturally in potatoes and can give a bitter taste to the protein product.^3−6^ GA levels can vary strongly between potato batches, which complicates the protein purification process and causes variations in the composition and quality of the final product. Therefore, ideally, the GA levels should be measured continuously during the manufacturing process so that the process settings in the factory can be adjusted continuously and in real time.

A sensor technology suited for real-time biochemical monitoring is biosensing by particle motion (BPM).^7−12^ BPM makes use of reversible affinity-based interactions between biofunctionalized particles and a biofunctionalized substrate. Transition rates are measured between bound and unbound states of particles, and these reflect the concentration of analyte in solution. Previously, we demonstrated the monitoring of nucleic acids, proteins, and small molecules using commercially available oligonucleotides, aptamers, and antibodies. However, suitable binder molecules are not always commercially available. To make the BPM real-time sensing technology widely applicable, strategies need to be designed for developing binder molecules and for testing and implementing these in the BPM platform. Here, we demonstrate the development of antibodies for real-time GA sensing in fluids from potatoes. We describe the design of the sensor and the development of its molecular components (analogues and antibodies), show GA monitoring results over long time spans, and discuss how the BPM technology can be applied for real-time monitoring of small molecules in industrial food processes.

## Materials and Methods

### Preparation of Solanidine Conjugates

Biotin-solanidine was prepared by mixing 2 mg azide-solanidine (S064550, TRC, Canada) dissolved in 200 μL of acetonitrile and 1.25 mg DBCO-PEG4-biotin (760749-5MG, Sigma-Aldrich) dissolved in 15 μL of acetonitrile. 20 μL of Milli-Q water was added to the mixture, and the whole mixture was incubated over 3 days until the solution was clear. LC–MS measurements of the reaction mixture confirmed that no DBCO-PEG4-biotin was left.

ssDNA-solanidine was prepared by mixing 5 mg of azide-solanidine (S064550, TRC, Canada) dissolved in 4 mL of methanol and 24 μL of DBCO-ssDNA (500 μM) (5′ DBCO—TGG TCT TAC CCC TGC CGC AC 3′). The reaction mixture was incubated at room temperature for over 48 h. The mixture was then dissolved in 0.17 mM NaCl in 98% ethanol, stored at −20 °C for 18 h, and subsequently centrifuged at 14,000 rpm for 15 min at 4 °C. The obtained pellet was washed with 0.17 mM NaCl in 98% ethanol, stored at −20 °C for 75 min, centrifuged at 14,000 rpm for 15 min at 4 °C, and washed three times with ethanol. Solanidine-ssDNA was obtained after evaporating the ethanol, redissolved in MilliQ water, and stored at −20 °C until further use. The identity of the conjugate was confirmed using agarose gel electrophoresis.

### Phage-Display Selection

Two phage-display libraries (Human scFv naive complexity ∼10^11^ and Llama VHH naive complexity ∼10^9^) were developed by AbSano, similarly to those described by Raats et al.^13^ Biotin-solanidine was used for antibody selection by panning three different scFv/VHH phage libraries separately using KingFisher (Thermo Fisher Scientific). Phages were harvested using standard NaCl/PEG precipitation and blocked for 1 h in 2 w/v % milk in PBS (MPBS; PBS: 137 mM NaCl, 2.7 mM KCl, 10 mM Na_2_HPO_4_, 1.8 mM KH_2_PO_4_, pH 7.4) at 4 °C. The blocked phages were incubated with control beads for deselection. Control beads were prepared by incubation of magnetic streptavidin beads (Dynabeads M-280 Streptavidin, Thermo Fisher Scientific, cat. #11206D) with DBCO-biotin at saturating concentrations according to the manufacturer’s instructions. Before addition to the phages, the beads were blocked in MPBS for 1 h. After a 1 h depletion, the phages were incubated with solanidine-functionalized beads. The beads were prepared in a similar way as described above for the control beads, now using a biotin-solanidine construct. After 1.5 h of selection, the beads were washed for 5 min. After one round of selection, all phages that bound to the beads were used to transfect *E. coli* TG-1 bacteria, for phage amplification for the next round of panning. Similar procedures were performed in rounds 2 and 3 of panning, with a decreasing number of beads and increased washing stringency (2–3 × 7 min). The infected bacteria were grown to single colonies for picking by RapidPick SP (Hudson Robotics). Polyclonal and individual phage clones of round 3 were tested for binding to solanidine on ELISA.

### ELISA Screening

Overnight, 96-well plates were coated with 1 μg/mL neutravidin in carbonate buffer at 4 °C. The next day, the plates were functionalized with 12 μM biotin-solanidine or DBCO-PEG4-biotin in PBS for 1 h at 37 °C, followed by a 1 h blocking step with MPBS at room temperature. Phages in culture supernatant diluted 1:1 in PBS were incubated for 1 h at room temperature. After washing (3× 0.05% Tween-20 in PBS (PBST) and 3× PBS), a 5000× dilution of anti-M13 antibody-HRP (Bio-Connect, cat. #11973-MM05T-H) in MPBS was added and incubated for 2 h before the detection of antigen-bound phages using TMB (Invitrogen, SB02) and H_2_SO_4_. The solanidine positive clones were sequenced for their scFv/VHH sequences to obtain unique phage binders. scFv/VHH from supernatants or purified materials in PBS were tested for binding (conc. 0.5–50 μg/mL diluted in PBST) in a similar way with detection using an *anti*-*c*-Myc monoclonal antibody (9E10) conjugated with HRP (Invitrogen MA1-81357).

### Recombinant (scFv)_2_-Fc and (VHH)_2_-Fc Antibody Expression

HEK293F cells (Invitrogen) were cultured in suspension in FreestyleTM 293 expression medium (Thermo Fisher Scientific, cat. #12338) at 37 °C, 110 rpm, 8% CO_2_, and 95% humidity. Cells were sub-cultured 3× per week at 0.3–0.5 million/mL in culture flasks. For counting and viability checks, an automated cell counter (Nexcelom Cellometer auto T4 Plus) was used, and cells were diluted 1:1 in 0.4% Trypan Blue (Thermo Fisher Scientific, cat. #15250061). Plasmids encoding for the recombinant antibodies were amplified in chemically competent *E. coli* XL1-blue bacteria and harvested using Mini/Midi/Maxi prep (Qiagen, cat. #27106, cat. #27191, cat. #12143; Sigma-Aldrich, cat. #NA0410). HEK293F cells (1 million/mL) were transfected with the FectoPro transfection reagent (PolyPlus-transfection, cat. #116) using 500 μg DNA/million cells for the (scFv)_2_-/(VHH)_2_-Fc (IgG1) format. After 2.5 h, transfection was boosted by the addition of 2 mM sodium butyrate. Cells were incubated at 37 °C, 110 rpm, 8% CO_2_, and 95% humidity. After 4–6 days, the cells were checked for viability, and the antibody was harvested when the viability dropped below 60%, as detected using automated cell counting. To harvest the antibody, the culture was first centrifuged at 300*g* for 10 min at 4 °C to remove the cells, and then at 4816*g* for 1 h at 4 °C to remove cell debris and aggregates. The supernatant, containing the recombinant antibodies in culture medium, was stored at 4 °C until further use. Antibody concentrations were assessed using Octet measurements (ForteBio Octet Red96) with a Protein A-coated sensor (Molecular Devices, cat. #18-5010) and a hIgG1 standard curve (Sartorius, cat. #18-1118).

### Recombinant (scFv)_2_-Fc and (VHH)_2_-Fc Antibody Purification

Recombinant (scFv)_2_-Fc and (VHH)_2_-Fc antibodies were purified using an Äkta Pure 25 system (Cytiva) equipped with a 1 mL HiTrap MabSelect SuRe column (Cytiva, cat. #11003493) and two 5 mL HiTrap Desalting columns with Sephadex G-25 resin (Cytiva, cat. #17140801) for tandem purification. In short, recombinant antibodies in cell supernatants were filtered using a 0.45 μm filter before manual loading on a sample loop, superloop, or using a pump. First, the antibodies were captured on the mAb Select SuRe column. The column was washed with 10 CV bind buffer (20 mM sodium phosphate, 150 mM NaCl, pH 7.2). The antibodies were eluted from the first column using elution buffer (100 mM sodium citrate, pH 3). The pure antibody was detected using UV absorbance at 280 nm and directly loaded on the desalting columns to exchange the buffer to PBS (pH 7.2) using 5 CV. Pure (scFv)_2_-Fc and (VHH)_2_-Fc antibodies were collected using a fraction collector based on the UV absorbance at 280 nm. The concentration of antibodies was determined by measuring the absorbance at 280 nm using a NanoDrop2000 spectrophotometer (Thermo Life Science) and calculating the extinction coefficient per (scFv)_2_-Fc and (VHH)_2_-Fc antibody. Antibody purity was determined using reducing and non-reducing SDS-PAGE analyses.

### Biotinylation of Antibodies

*anti*-Solanidine antibodies were dissolved in Dulbecco’s phosphate buffered saline (PBS) at a concentration of around 1 mg/mL. EZlink NHS-PEG4-biotin (Thermo Scientific) was prepared according to the manufacturers’ instructions and added to the antibodies. The excess NHS-PEG4-biotin was removed using Amicon Ultra 0.5 mL centrifugal filters (10 K) at 15,000 rpm for 10 min at 4 °C. The biotinylated antibodies were then stored in PBS at 4 °C until further use.

### Functionalization of Particles

For the preparation of particles for antibody screening in microtiter plates, streptavidin-coated particles (10 mg/mL, Dynabeads MyOne Streptavidin C1, Thermo Scientific) were mixed with 10 μM biotin-modified capture ssDNA (5′ biotin—GTG CGG CAG GGG TAA GAC CA) in PBS in a 1:2 (particles/ssDNA) volume ratio. After an incubation period of 30 min in a rotating fin (VWR, The Netherlands), the particles were blocked with 100 μM 1 kDa mPEG-biotin (PG1-BN-1k, Nanocs) for 15 min while rotating. The particles were washed twice with 500 μL of PBS with 0.05% (v/v) Tween-20 (PBST) and reconstituted in PBS-BSA. Particles were provided with analogues by adding solanidine-ssDNA molecules (1 μM) and incubating this mixture for 1 h at room temperature while rotating, allowing it to hybridize with the particle-side capture ssDNA. The particles were again washed twice with 500 μL of PBST and reconstituted in PBS-BSA, after which the mixture was sonicated in an ultrasonic bath (Branson Ultrasonics) for 1 min.

For the preparation of particles for glycoalkaloid detection in flow chambers, streptavidin-coated particles were mixed with a 250 nM biotinylated *anti*-solanidine antibody in a 1:1 (antibody/particles) volume ratio and left to incubate for 30 min while rotating at room temperature. The particles were blocked afterward with 10 μM of polyT (5′ biotin—TTT TTT TTT TTT TTT T 3′) for 45 min while rotating at room temperature. The particles were washed twice with 500 μL of PBST, reconstituted in PBS supplemented with 0.5 M NaCl (PBS–NaCl), and sonicated in an ultrasonic bath for 1 min.

### Preparation of Microtiter Well Plates for Antibody Screening

*anti*-Solanidine antibodies were dissolved in 50 mM sodium carbonate buffer (pH 10). 50 μL of the antibody mixture was added to Nunc MaxiSorp FlatBottom 96-well plates (Thermo Scientific) and allowed to incubate for over 16 h at 4 °C. The wells were subsequently blocked with 100 μL PBS with 1% (w/v) bovine serum albumin (PBS-BSA) at room temperature for at least 1 h. The supernatant was then removed, after which 50 μL of the particle mixture was added and incubated for 1 h.

### Flow Chamber Preparation for Glycoalkaloid Detection

A poly(l-lysine)-*grafted* poly(ethylene glycol) (PLL-*g*-PEG) polymer mixture was prepared using 0.45 mg/mL PLL-*g*-PEG and 0.05 mg/mL PLL-*g*-PEG-N_3_, as described by Lin et al.^8^ Fluidic slides (μ-Slide II 3in1, ibidi GmbH) were pre-cleaned by 10 min of sonication in Milli-Q water. The substrate was dried with a nitrogen stream and subsequently ozone-cleaned (UV Ozone Cleaner, Novascan) for 30 min. The slides were sealed with clear polyolefin tape (Thermo Scientific), after which the PLL-*g*-PEG/PLL-*g*-PEG-N_3_ mixture was injected immediately to the flow chamber and left for 3 h at room temperature to allow the polymer to self-assemble onto the negatively charged substrate. Free polymer was removed by extracting the solution out of the flow chamber. The dsDNA tether (5′ DBCO—GGT TAG CAG CCT GTT TCA AAA CCT GGG GGT GAG TGT CAC GCC AAT TCA GCG CAT CGT TCT GTC GGG AGA GAA TGG TCT GAA AAT CGA TAT CCA CGT CAT TAT CCC GTA CGA AGG TCT TTC TGG TGA TCA GAT GGG GCA GAT AGA AAA AAT ATT CAA AGT GGT GTA CCC AGT AGA CGA TCA TCA CTT CAA GGT TAT ACT GCA CTA TGG CAC CCT CGT TAT CG 3′, 3′ CCA ATC GTC GGA CAA AGT TTT GGA CCC CCA CTC ACA GTG CGG TTA AGT CGC GTA GCA AGA CAG CCC TCT CTT ACC AGA CTT TTA GCT ATA GGT GCA GTA ATA GGG CAT GCT TCC AGA AAG ACC ACT AGT CTA CCC CGT CTA TCT TTT TTA TAA GTT TCA CCA CAT GGG TCA TCT GCT AGT AGT GAA GTT CCA ATA TGA CGT GAT ACC GTG GGA GCA ATA GC—biotin 5′) was prepared as described by Yan et al.,^9^ diluted in PBS–NaCl to a concentration of 0.5 nM, and injected into the flow chamber. After an incubation period of approximately 15 h at room temperature, the free dsDNA tether was removed and the flow chamber was provided with DBCO-modified capture ssDNA (5′ DBCO—GTG CGG CAG GGG TAA GAC CA). The prepared chambers were stored for at least 3 days before use.

On the day of use, antibody-functionalized particles were injected into the flow chamber (Harvard pump 11 Elite, 100 μL/min for 2 min for all steps unless indicated otherwise). The particles were allowed to interact with the biotin groups on the substrate-side dsDNA tether for approximately 12 min before flipping the flow cell and letting unbound particles sediment away from the functionalized surface. 100 μM of 1 kDa mPEG-biotin was then added to block the particles for 15 min. The system was activated by adding 2.5 to 5 nM of solanine-ssDNA conjugates and allowing it to hybridize with the substrate-side capture ssDNA. Particles were tracked continuously to monitor the switching activity during the incubation period (around 5 to 15 min), which was stopped by removing the excess solanidine-ssDNA by aspirating the flow chamber with PBS–NaCl. Samples containing GAs were prepared beforehand and injected into the flow cell chamber, after which the particle motions were recorded for 3 min in the absence of flow.

### Preparation of Potato Fruit Juice Samples

Two raw potato fruit juice (PFJ) samples with high (937 mg/L α-solanine, 469 mg/L α-chaconine) and low (29 mg/L α-solanine, 13 mg/L α-chaconine) TGA levels were kindly provided by Avebe and characterized on glycoalkaloid content using HPLC.^15^ Samples were centrifuged at 10,000 rpm for 4 min, after which the supernatant was carefully aspirated with a pipette and transferred to Protein LoBind tubes (Eppendorf). Reference samples were prepared by mixing both PFJ samples to achieve final TGA concentrations of 8.16, 2.62, 1.82, 1.03, 0.65, and 0.24 μM. The test samples were prepared in a similar manner. All reference samples were diluted 200-fold in PBS–NaCl. The test samples were diluted either 100-, 200-, 400-, or 800-fold in PBS–NaCl. The exact dilution protocol can be found in Table 1. Both reference and test samples were vortexed for 30 s and sonicated for 2 min in a sonication bath before injecting into the measurement chamber.

**Table 1:** Preparation of Potato Fruit Juice Test Samples Containing Native GAs^a^

sample	TGA (μM)	dilution factor	TGA after dilution (μM)
A	1394	400	3.49
		800	1.72
B	1045	200	5.23
		400	2.61
C	167	100	0.17
D	64	100	0.64

a TGA refers to total glycoalkaloid.

### Image Recording and Data Analysis

Videos of free particles in microtiter wells were made on a Nikon Ti confocal microscope (Nikon Instruments Europe BV, The Netherlands) at a magnification of 20× using an iXon Ultra 897 EMCCD camera (Andor, Belfast, UK) in bright-field illumination conditions, using Nikon NIS-Element imaging software. The positions of the particles were recorded in a field of view of 410 × 410 μm^2^ at a frame rate of 33 Hz with an exposure time of 5 ms. The particles were localized using phasor-based localization, after which the *x*–*y* trajectories were used to calculate the mean squared displacement over time using a sliding window algorithm. To discriminate between unbound and bound states of particles, a threshold on the calculated diffusion coefficient was set at 0.12 μm^2^/s.

Tracking of tethered particles in flow chambers was done on a custom-made optical setup at a total magnification of 10× using a Grasshopper camera (Point Grey Research Grasshopper3 GS3-U3-23S6M, 1920 × 1200, pixel format: 8 raw, gain 10) in bright field illumination conditions. The positions of the particles were recorded in a field of view of 659 × 493 μm^2^ at a frame rate of 30 Hz with an exposure time of 5 ms. The particles were localized using phasor-based localization, after which the *x*–*y* trajectories were used to detect particle switching events using a change-point detection algorithm, which has been described by Bergkamp et al.^14^

## Results

**Figure 1:**
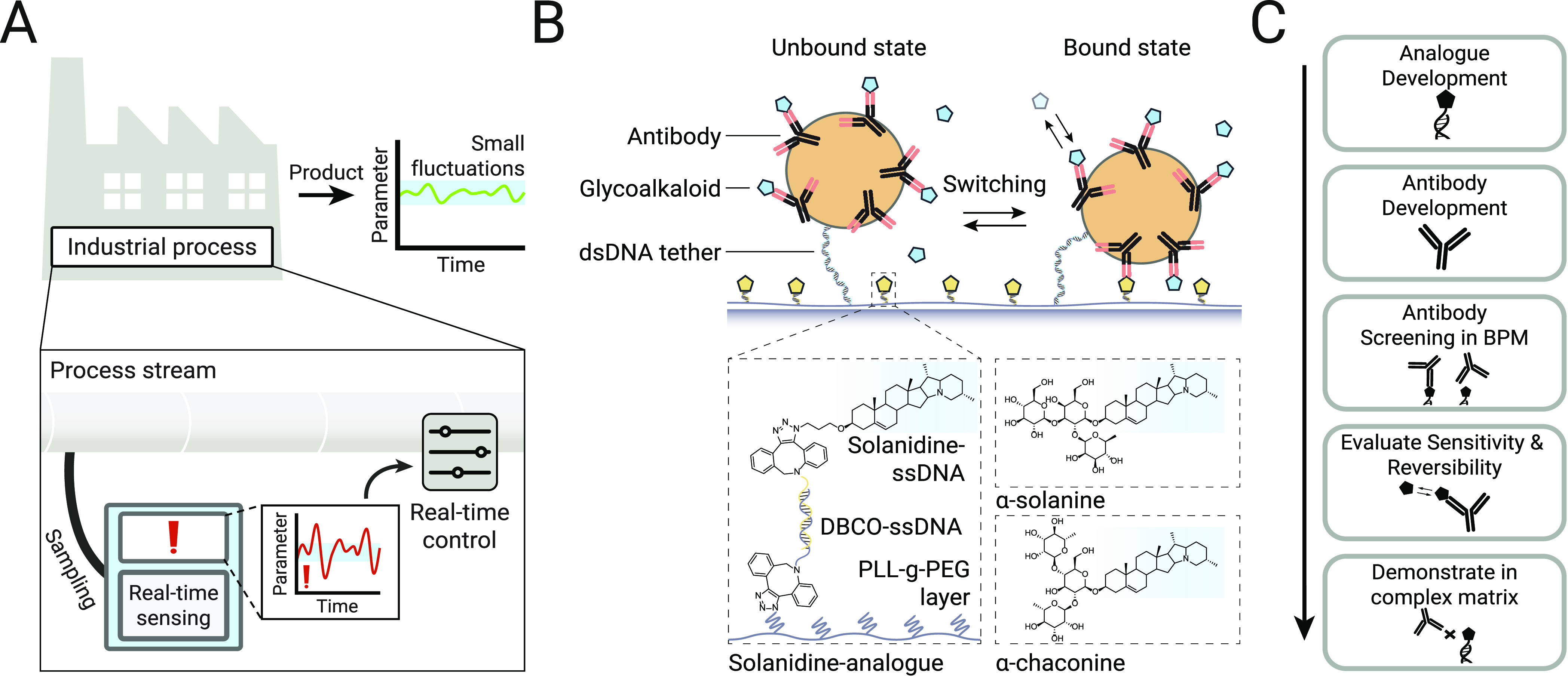
Continuous monitoring of GAs using BPM. (A) Sketch of continuous monitoring for measurement and control in industrial food processes. A real-time sensing system takes samples from a process stream to continuously measure the analytes of interest for control of the production process. The resulting product has well-defined quality with small variability. (B) Molecular design of the GA BPM sensor. The sensor surface is coated with a low-fouling PLL-*g*-PEG polymer layer and provided with analyte-analogue molecules. Particles are tethered to the surface via double-stranded DNA. The particles are functionalized with recombinant antibodies that reversibly bind to the analogue and to the analyte molecules. The switching events between bound and unbound states are detected by tracking the movement of the particles using video microscopy. The two major potato GAs are shown: α-solanine and α-chaconine. The common solanidine moiety is highlighted in blue. (C) Stepwise approach to develop the competitive GA biosensor.

**Figure 2:**
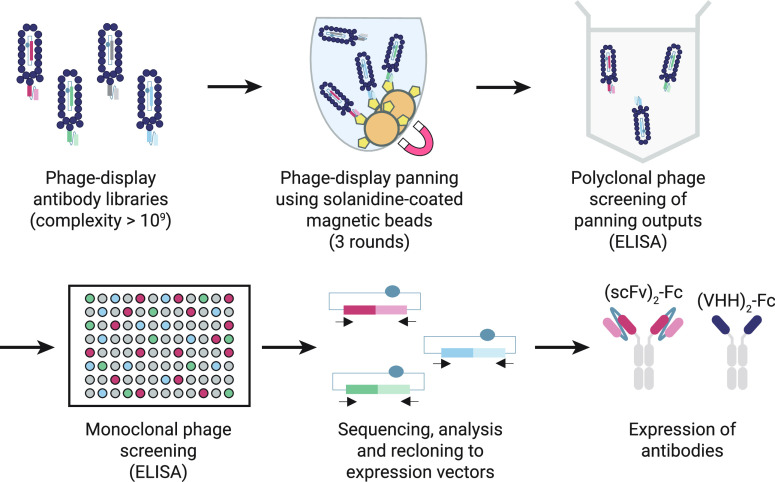
Antibody development. Flow chart of the antibody development pipeline. Phage libraries were used for panning against the solanidine analogue. The polyclonal phage outputs of each panning round were screened for reactivity toward the solanidine analogue. Of the positive samples, monoclonal phages were generated and selected. After sequencing of the phages, monoclonal antibodies were generated and expressed.

Figure 1A sketches how a continuous biosensor can be used for industrial process control. Samples are continuously taken from a manufacturing line and measured in a biosensor that continuously gives data about analyte concentrations. The generated stream of real-time data is used to adapt the manufacturing process in order to reduce fluctuations in key output parameters of the production process. Such a measurement and control application can function well when the sampling and measurement are fast with respect to the typical timescales of the fluctuations in the manufacturing process. Figure 1B shows the sensor studied in this paper based on BPM (Section S1).^7−12^ Streptavidin-coated particles (1 μm in diameter) are functionalized with biotinylated *anti*-solanidine antibodies. The particles are bound to a substrate using a double-stranded DNA tether (dsDNA, around 50 nm in length), which is modified with biotin and dibenzylcyclooctyne (DBCO) on either end. The substrate is coated with poly(l-lysine)-*grafted*-poly(ethylene glycol) (PLL-*g*-PEG) and PLL-*g*-PEG-azide that provide low-fouling properties of the surface and allow covalent coupling of DBCO-modified molecules, including the dsDNA tether. The polymer coating is functionalized with DBCO-single stranded DNA (ssDNA) molecules, to which solanidine-ssDNA conjugates are hybridized. The solanidine-ssDNA conjugates are substrate-side binders that act as analyte-analogue molecules. The sensor is sensitive to both α-solanine and α-chaconine, the two major GAs in potatoes, as each of these molecules contains a solanidine moiety.^3−6^ When an antibody-functionalized particle binds to analyte-analogue molecules on the substrate, the particle changes from a state with high motional freedom to a state with very little motional freedom, i.e., from an unbound to a bound state (Figure 1B). The state transitions of particles are detected using widefield optical microscopy. The probability that a particle becomes bound to the surface is decreased in the presence of the analyte, as these bind to the antibodies and reduce the probability that a particle binds to analyte-analogue molecules on the substrate. This leads to a decrease in the binding frequency of the particles, which is used as a measure for the analyte concentration (Figure S1B). The sensor design relies on *anti*-solanidine antibodies; however, these were not commercially available. Therefore, antibodies were developed in this study using phage-display technology, as shown in Figure 2. Pre-existing phage-display scFv and VHH libraries were used for the selection of novel recombinant antibodies. Biotin-solanidine conjugates were synthesized by coupling azide-solanidine to DBCO-(PEG)_4_-biotin for use during phage screening. Human scFv and Llama VHH phage-display libraries were first separately subjected to three rounds of panning on solanidine-coated magnetic beads. The output phages were screened for binding to biotin-solanidine by ELISA. Binding phages were identified by sequencing and re-cloned into an (scFv)_2_-Fc/(VHH)_2_-Fc expression vector to generate recombinant antibodies. A total of 36 recombinant antibodies were expressed in a mammalian expression system and tested for binding to biotin-solanidine by ELISA. This resulted in 13 recombinant antibody candidates, which were selected for further testing to investigate their use in the BPM sensor platform (Section S2).

### *Anti*-solanidine Antibody Selection in BPM

We tested the 13 recombinant antibodies from the phage-display libraries by studying antibody–analogue interactions in microtiter well-plates using f-BPM, which is a BPM sensor variant with particles freely hovering over a sensing surface.^12^ The Brownian motion of freely diffusing particles is tracked to determine the diffusivity of the particles as a function of time. A high diffusivity corresponds to free Brownian motion, while a low diffusivity corresponds to confined Brownian motion caused by temporal particle–surface interactions. The fraction of bound particles (i.e., with low Brownian motion) is used as a measure of binding between antibodies and solanidine.

**Figure 3:**
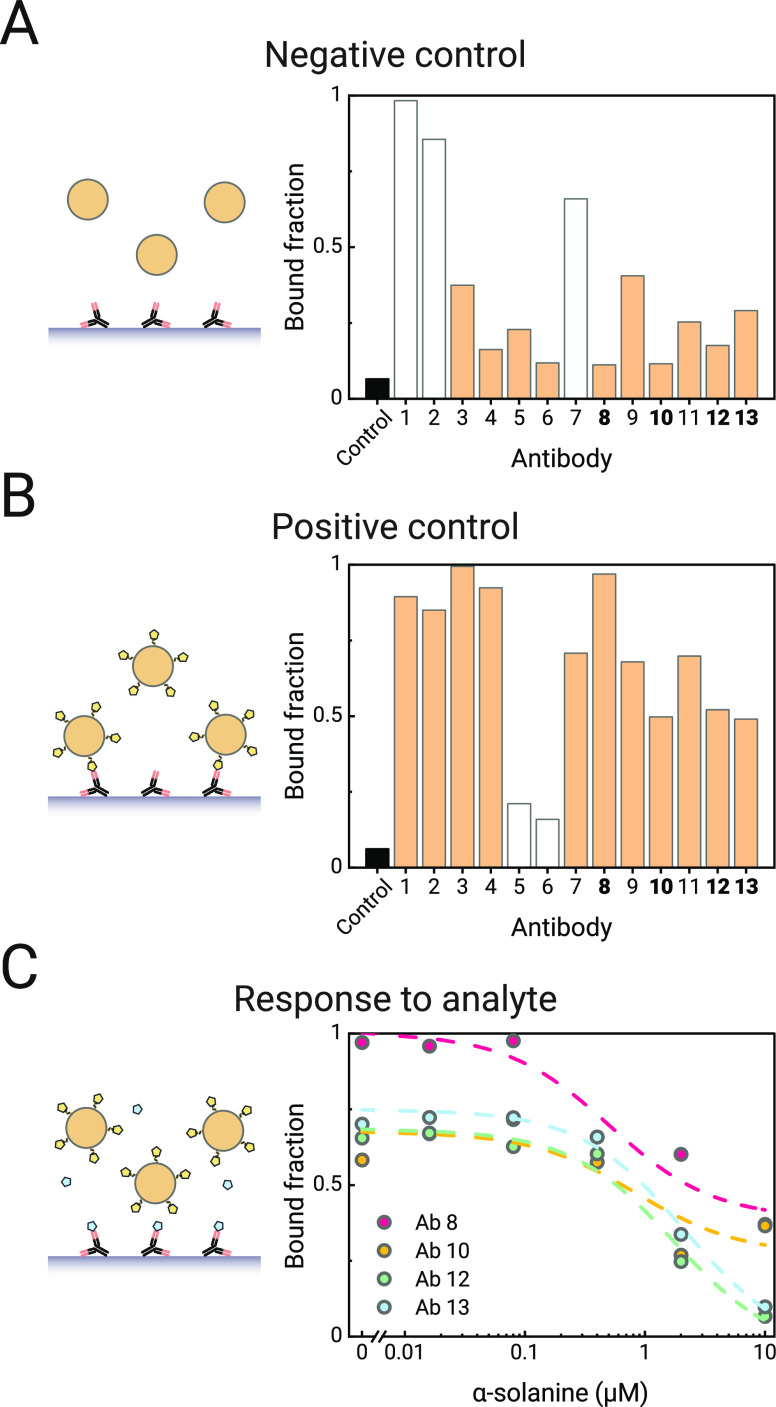
Screening of recombinant *anti*-solanidine antibodies using an f-BPM assay in a microtiter well-plate. Antibodies were immobilized through physisorption on a well plate surface. The Brownian motion of particles hovering over a biofunctionalized surface is recorded. The fraction of unbound and bound particles is derived from the observed diffusivities of the particles. (A) Negative control experiment. Antibodies were immobilized at a 50 nM concentration. Orange bars highlight antibodies with low non-specific binding. (B) Positive control experiment. Antibodies were immobilized at a 50 nM concentration. Particles were functionalized with biotin-coupled solanidine-analogue molecules at a concentration of 10 nM. Orange bars highlight antibodies with binding to solanidine-coated particles. (C) Response to the addition of α-solanine. Antibodies were immobilized at a 6.25 nM concentration. Particles were functionalized with biotin-coupled solanidine-analogue molecules at a concentration of 5 nM. α-Solanine was added to the particles prior to pipetting the solution into the microtiter wells.

Figure 3 shows how biofunctionalized particles interact with a microtiter plate surface coated with physisorbed recombinant antibodies. The interaction was quantified by measuring the bound fraction, i.e., the fraction of particles bound to the surface. Negative control experiments were performed, with particles having only streptavidin on their surface (Figure 3A). Positive control experiments were performed with particles having solanidine-ssDNA conjugates hybridized to complementary biotin-ssDNA conjugates that were coupled to particles via streptavidin-biotin bonds (Figure 3B). Antibodies with good binding properties are characterized by low non-specific binding (i.e., low bound fraction in the negative control experiment, Figure 3A) and specific interactions with the analogue molecule (i.e., high bound fraction in the positive control experiment, Figure 3B). Only a few antibodies did not show these characteristics; these either bound non-specifically with the particles (antibodies 1 and 2), or they did not show binding to the solanidine-analogue (antibodies 5 and 6). The competition assay performance was tested by adding α-solanine to determine if the analyte was able to compete with solanidine-analogue molecules for binding to the antibodies. Figure 3C shows how the bound fraction depends on the analyte concentration for selected recombinant antibodies. After immobilization on the well-plate substrate, the antibodies were incubated with analogue-functionalized particles and the analyte simultaneously. The data show that free α-solanine is indeed able to compete for binding sites on the antibodies and reduce the fraction of particles in the bound state, with effectiveness in the submicromolar to micromolar range. Other antibodies, including those that did not show a competitive response to free α-solanine, are shown in Figure S3.

### Sensor Performance

**Figure 4:**
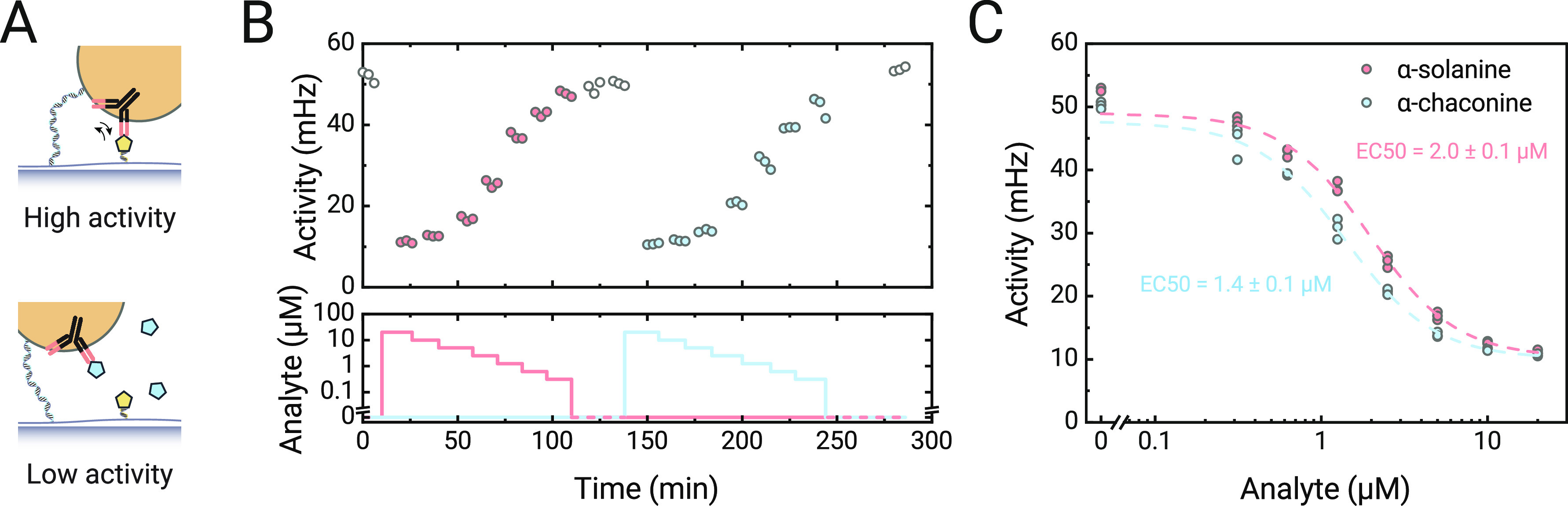
Continuous monitoring of α-solanine and α-chaconine in a buffer solution measured in a t-BPM GA sensor. (A) Sketch of reversible particle switching in the absence of the analyte (top) and in the presence of the analyte (bottom). Antibodies are sketched in black, solanidine conjugates in yellow, and glycoalkaloid analytes in blue. (B) Measured switching activity over a period of 5 h. The bottom panel shows the supply of the fluid with a time series of analyte concentrations into the flow cell, first with α-solanine (red line), and thereafter with α-chaconine (blue line). (C) Dose–response curve for α-solanine (red) and α-chaconine (blue) obtained with data from panel (b). The data points were fitted with a sigmoidal curve , resulting in EC50 values of 2.0 ± 0.1 and 1.4 ± 0.1 μM (fitted value ± standard error of the fitted value) for α-solanine and α-chaconine, respectively.

The sensitivity and reversibility of the GA sensor were studied in flow cell experiments with a BPM sensor with tethered particles (Figure 4). Biotinylated *anti*-solanidine antibodies (antibody 12, scFv-Fc antibody) were attached to streptavidin-coated microparticles that were tethered to the sensor surface using double-stranded DNA. The solanidine-ssDNA conjugate was coupled to substrate-side complementary ssDNA strands. The sensor response relies on reversible binding between the solanidine-analogue molecules on the sensor surface and recombinant antibodies on the particles, as sketched in Figure 4A. The particles function as transducers, whose motion characteristics change depending on the analyte concentration. The motion characteristics are measured optically without requiring any reagents, i.e., the sensor does not consume nor produce any reagents during the measurements.

Figure 4B shows the continuous monitoring results of α-solanine and α-chaconine. Concentration series of both GAs were added to the same flow cell from high to low concentrations, and several blank measurements were included. The top panel shows the response of the sensor to changes in the analyte concentration. The sensor response was corrected for drift over time (Section S4).^8^ The activity of the sensor correlates inversely with both the α-solanine and α-chaconine concentrations. In the absence of the analyte, the activity is high due to the high frequency of binding events of particles with the analyte-analogue molecules on the substrate. The activity decreases upon the addition of analyte molecules, as these bind to the *anti*-solanidine antibodies. The reversibility of the sensor is demonstrated by the consistent activity values of about 55 mHz when fluid without GA was flown into the flow cell (at *t* = 0 min, at *t* = 125 min, and at *t* = 280 min). The reversibility is enabled by the relatively high dissociation rate constant of the antibody, allowing the release and removal of analyte molecules when fluid with a lower analyte concentration enters the flow cell.

Figure 4C shows the same data but now plotted as a dose–response curve with a sigmoidal fit. The two curves correspond to the concentration series of the two GAs that were added to the flow cell. The sensor is shown to be sensitive in the low micromolar range for both GAs, with a dynamic range that spans roughly 2 orders of magnitude. The measured EC50 values of the two GAs (EC50_α-solanine_ = 2.0 ± 0.1 μM and EC50_α-chaconine_ = 1.4 ± 0.1 μM) are close to each other, indicating that the antibodies have similar affinities to both analyte molecules.

The underlying molecular interactions can be further analyzed by studying the lifetimes of the unbound and bound states measured in the presence of α-solanine and α-chaconine (Figure S4). Particle unbound-state lifetimes reflect the effective association rate between the particle and the substrate. The association is governed by the antibody density on the particle, the analogue density on the substrate, and the analyte concentration in solution. Particle bound-state lifetimes reflect the effective dissociation rate of particles from the substrate. In the regime of single molecular bonds between the particle and the substrate, the bound-state lifetimes are governed by the dissociation rate constant of the molecular bond between the *anti*-solanidine antibody on the particle and the solanidine-analogue on the substrate. The distribution functions of measured unbound-state lifetimes in the presence of α-solanine (Figure S4A) and α-chaconine (Figure S4B) could be fitted with a multi-exponential function. This multi-exponential function relates to interparticle variations in binder densities, leading to interparticle variabilities in unbound-state lifetimes.^7,15^Figure S4C shows that the characteristic unbound-state lifetimes for both GAs increase similarly as a function of the concentration. This implies that the occupancy of antibody-binding sites increases similarly for both GAs. Characteristic bound-state lifetimes were obtained by fitting the distribution functions of measured bound-state lifetimes in the presence of α-solanine (Figure S4D) and α-chaconine (Figure S4E). Figure S4F shows that the bound-state lifetimes related to specific bonds are independent of the analyte concentration, indicating that the bound states are caused by the single-molecular interactions between the antibody and analyte-analogue. The characteristic bound-state lifetime was found to be about 30 s, corresponding to an effective dissociation rate *k*_off_ of 0.03 s^–1^.

**Figure 5:**
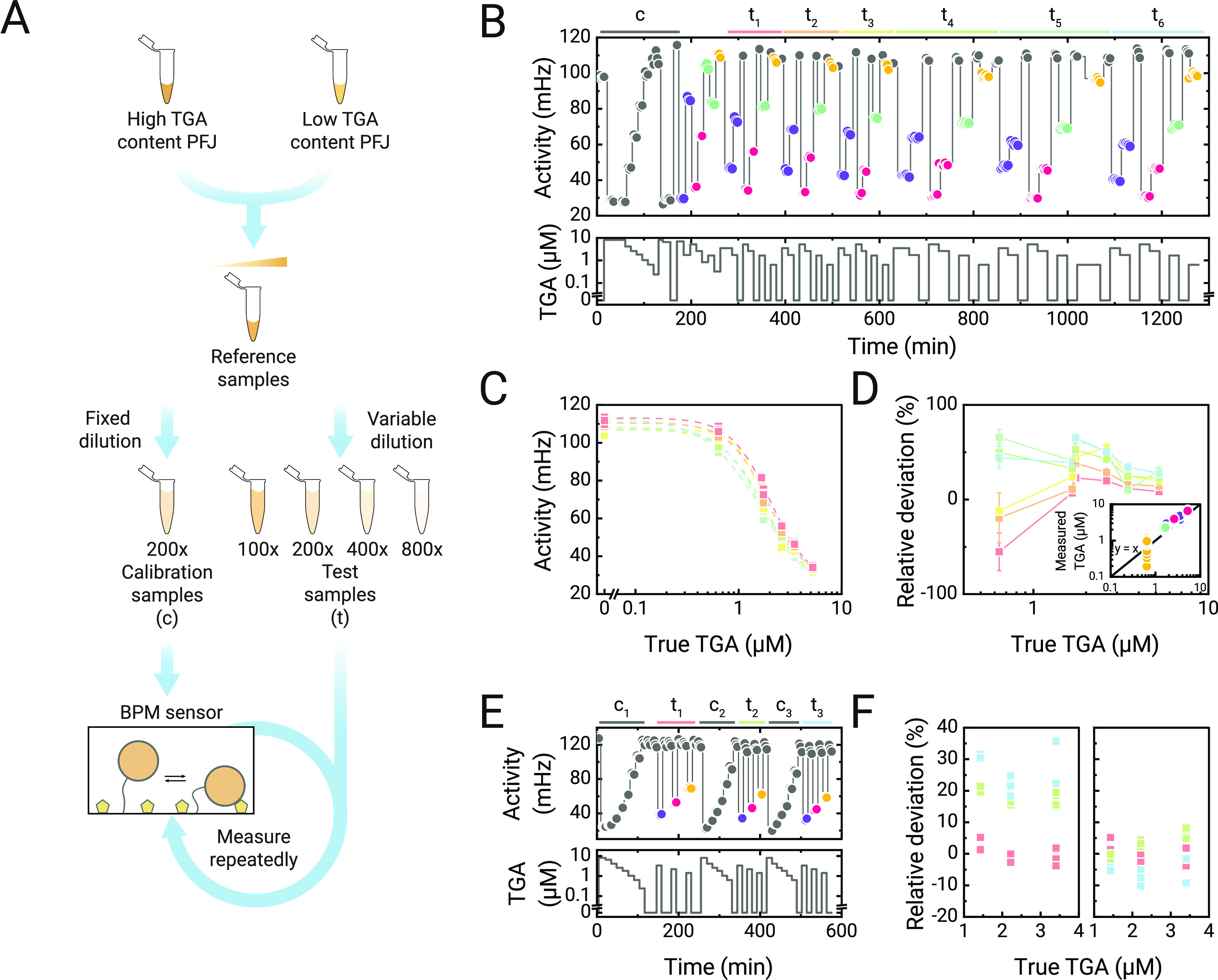
Continuous monitoring of native GA in potato fruit juice (PFJ) on a BPM sensor. (A) Preparation of PFJ samples containing varying levels of total glycoalkaloid (TGA). Samples with a high and low level of TGA were obtained. These were characterized on TGA levels using HPLC and thereafter mixed in different ratios to create PFJ samples with different TGA levels. Two series of samples were prepared, a calibration series and a test series. Calibration samples were diluted 200-fold prior to measurement in the sensor. Test samples were diluted 100- to 800-fold prior to measurement. (B) Measured switching activity over a total period of more than 20 h. The bottom panel shows the TGA level over time, and the top panel shows the measured response of the sensor. The sensor response was corrected for drift (Section S4). The top bars indicate different measurement blocks, in which first a single calibration series was measured (gray, labeled with c), followed by six blocks of the test samples (colored, labeled t_1_ to t_6_). The colored data points in the top panel correspond to samples with different reference TGA levels (1394 μM, purple, measured with 400- and 800-fold dilution; 1045 μM, pink, 200- and 400-fold dilution; 167 μM, 100-fold dilution, mint; 64 μM, 100-fold dilution, yellow). The lines serve to guide the eye. (C) Dose–response curves for TGA content in native PFJ, based on data points shown in panel (B). The colored curves correspond to the measurement blocks from panel (B). The TGA concentration shown on the *x*-axis corresponds to the concentrations including the applied dilution factors. (D) Concentration deviations of the BPM sensor based on the data points in panel (B). The colored curves correspond to the measurement blocks from panel (B). The relative deviations of the concentration measurement on the BPM sensor were determined by comparing the results to the known TGA levels of the reference samples, as determined by HPLC. The measured TGA levels were calculated using a sigmoidal curve fit of the measured calibration curve, determined using the calibration samples. The TGA concentration shown on the *x*-axis corresponds to the concentrations including the applied dilution factors. The lines serve to guide the eye, and error bars indicate the standard deviation. The inset shows the measured TGA levels of the BPM sensor compared to the reference HPLC method. The colors of the data points correspond to the colors of the data points in panel (B). (E) Study of the BPM sensor with three calibration series. The bottom panel shows the TGA level over time, and the top panel shows the measured response of the sensor. The top bars indicate different measurement blocks, with three calibration series (gray, labeled c_1_, c_2_, and c_3_), and three measurement blocks of test samples (colored, labeled t_1_, t_2_, and t_3_). (F) Concentration deviations of the BPM sensor when using only a single calibration (only c_1_) and when using all three calibrations (c_1_, c_2_, and c_3_). Colors correspond to the measurement blocks from panel (E).

To investigate the performance of the BPM sensor with complex biological samples, the sensor was subjected to multiple samples of potato fruit juice (PFJ) containing native GAs over a period of 20 h (Figure 5). PFJ is a by-product of potato starch production and is the main source of potato proteins for human consumption. PFJ contains 15–20 mg/mL of soluble protein, in addition to other substances such as minerals, organic acids, phenolic compounds, amino acids, and GAs.^4,5,16^ Quantitative analysis of GAs is currently performed with HPLC and LC–MS methods, which require extensive sample preparation protocols to extract GAs from potato proteins within the PFJ.^17−19^ The level of GAs in potatoes is commonly reported as a total glycoalkaloid (TGA) level, which is the sum of measured α-solanine and α-chaconine concentrations.

PFJ samples with different levels of TGA were obtained by mixing PFJ samples with a high and low TGA content at different ratios to obtain a range of concentrations that were representative for industrially processed PFJ (Figure 5A). The performance of the BPM sensor over time was evaluated by exposing the sensor to a series of PFJ samples with different GA concentrations. Prior to insertion in the sensor, the samples were centrifuged to remove insoluble components and then diluted in order to bring TGA levels into the measurable range (concentrations and dilution factors can be found in the Materials and Methods section). The sequence of steps, i.e., sample preparation (around 4 min), sample injection (2 min), and image recording (3 min), resulted in a total time-to-result of about 10 min per sample.

Figure 5B shows the continuous monitoring results of native TGA in PFJ over a measurement period of over 20 h. Multiple series of PFJ samples were supplied to the same sensor. A calibration series (*t* = 0–170 min) was followed by six repeated measurement blocks of test samples. Blank measurements were used to correct for the sensor signal drift. Figure 5C shows the measured activity signals as a function of the TGA concentration for the six series of test samples. The curves are very similar, with a slight shift toward lower concentrations as a function of time. The data were fitted with a sigmoidal function, providing EC50 values of 2.0 ± 0.1 (red), 1.9 ± 0.1 (orange), 1.8 ± 0.1 (green), 1.6 ± 0.2 (turquoise), and 1.6 ± 0.1 (blue) μM.

A consequence of the shift of the curve over the period of 20 h is that measurements of unknown concentrations become less and less accurate over time. The deviation between measured concentrations and applied concentrations is studied in Figure 5D. Here, TGA concentrations of test samples were determined based on the initial calibration curve, the measured BPM signal, and the known dilution factors. The colors of the square data points correspond to the colors of the six test series (t_1_ to t_6_) in panel B. The samples with the lowest TGA concentration (inset, yellow data points) show the largest deviations from the true TGA concentration and the largest spread, pointing to inaccuracy and imprecision. Over time, the relative deviations increase, meaning that the sensor overestimates the TGA concentration in the test samples because of the gradual changes of the dose–response curve as a function of time, as shown in panel C.

To reduce the deviations of the TGA results of the BPM sensor, a testing protocol was developed with repeated calibrations to better correct for changes of the sensor characteristics. Figure 5E shows a sequence with three calibrations and three series of test samples. Figure 5F summarizes the relative deviations when making use of only a single calibration (left) and when making use of three calibrations (right) prior to measuring the test samples. The data show that more frequent calibrations strongly reduce the deviations of the concentration readings of the BPM sensor. The measured concentrations are spread in the range of −10 to +10% compared to the true TGA concentration, indicating that the relative deviations related to accuracy and precision are smaller than 10%.

## Discussion and Conclusions

We have presented a methodology to design an affinity-based sensor for the continuous monitoring of small molecules in industrial food processes, exemplified by the measurement of GAs in PFJ. Antibodies were developed from a phage-display library and screened using BPM with free particle motion. Thereafter, the antibodies were implemented in a tethered BPM sensor, and the sensor was studied in terms of sensitivity, reversibility, and stability.

Conventional laboratory analysis methods such as high-performance liquid chromatography and mass spectrometry are complicated and slow due to the required sample pretreatment steps and the measurement runtime, which make the methods unsuited for real-time industrial process control. Optical spectroscopic methods such as Raman and infrared analyses are much faster but lack sensitivity and specificity for the measurement of low-concentration substances in biological fluids that are variable and complex. Affinity-based biosensors may provide a solution because they can be sensitive, specific, and fast. However, to be suited for real-time measurement and control in industrial processes, the biosensing technology needs to (1) achieve a response time that matches the temporal fluctuations in the industrial process and (2) demonstrate continuous sensor operation over prolonged periods of time, with suitable sensitivity and specificity for the biomolecule and matrix of interest.

The results in this paper demonstrate that BPM biosensing of small molecules in an industrial food sample matrix has a total sample-to-answer response time of 5–10 min, which is achieved because the sample handling steps are simple and the sensor exposure to sample fluid is brief. The sensor is reversible due to the effective dissociation rate of the antibody–analyte interactions and allows the testing of a series of samples on a single BPM sensor over a long time span (20 h). Concentration readings with deviations below 10% can be achieved in the low micromolar range when intermittent calibrations are applied. In follow-up work, we will optimize the calibration methods of the BPM sensor and study molecular coupling strategies to further extend the operational lifetime of the sensor. Moreover, we will characterize the analytical performance in more detail, including the accuracy, precision, and sources of variation over short and long timescales.

In summary, we have shown that the developed BPM sensor is analytically suited for monitoring GA levels in samples from a potato processing stream. This paves the way for implementation of the real-time immunosensor in the industrial manufacturing of potato protein and thereafter also in other industrial food processes that can benefit from real-time process control based on continuous small-molecule measurements.

## Supporting Information Available

Measurement principle of BPM, recombinant antibodies obtained from phage-display libraries, f-BPM microtiter screening for detection molecules, activity drift correction, and analysis of state lifetimes for different concentrations of α-solanine and α-chaconine (PDF)
