# Supplementary material for: Real-Time Immunosensor for Small-Molecule Monitoring in Industrial Food Processes
Source: Anal Chem. 2023 May 13;95(20):7950–9. doi: 10.1021/acs.analchem.3c00628 (PMC10209984; doi:10.1021/acs.analchem.3c00628)
Supplement: Supplementary file 1 — ac3c00628_si_001.pdf [file ac3c00628_si_001.pdf]

# Supporting information

## Real-time immunosensor for small molecule monitoring in industrial food processes

Chris Vu[1,2], Yu-Ting Lin[3], Stijn R. R. Haenen[3], Julia Marschall[4], Annemarie Hummel[4], Simone F. A. Wouters[4], Jos M. H. Raats[4], Arthur M. de Jong[2,5], Junhong Yan[3] and Menno W. J. Prins[1,2,3,5]

[1]Department of Biomedical Engineering, Eindhoven University of Technology, 5612 AZ, Eindhoven, the Netherlands.
[2]Institute for Complex Molecular Systems (ICMS), Eindhoven University of Technology, 5612 AZ, Eindhoven, the Netherlands.
[3]Helia Biomonitoring, 5612 AR, Eindhoven, the Netherlands.
[4]AbSano, 5349 AB, Oss, the Netherlands.
[5]Department of Applied Physics and Science Education, Eindhoven University of Technology, 5612 AZ, Eindhoven, the Netherlands.

## Contents

S1. Measurement principle of Biosensing by Particle Motion (BPM) for a competitive format ...... S1

S2. Selected recombinant antibodies from phage display libraries after ELISA selection................ S2

S3. Response of selected anti-solanidine antibodies upon the addition of α-solanine using fBPM microtiter screening ........................................................................................................... S3

S4. Correction of raw measurement data of α-solanine and α-chaconine .................................... S4

S5. Analysis of state lifetimes in the presence of α-solanine and α-chaconine .............................. S6

## S1. Measurement principle of Biosensing by Particle Motion (BPM) for a competitive format

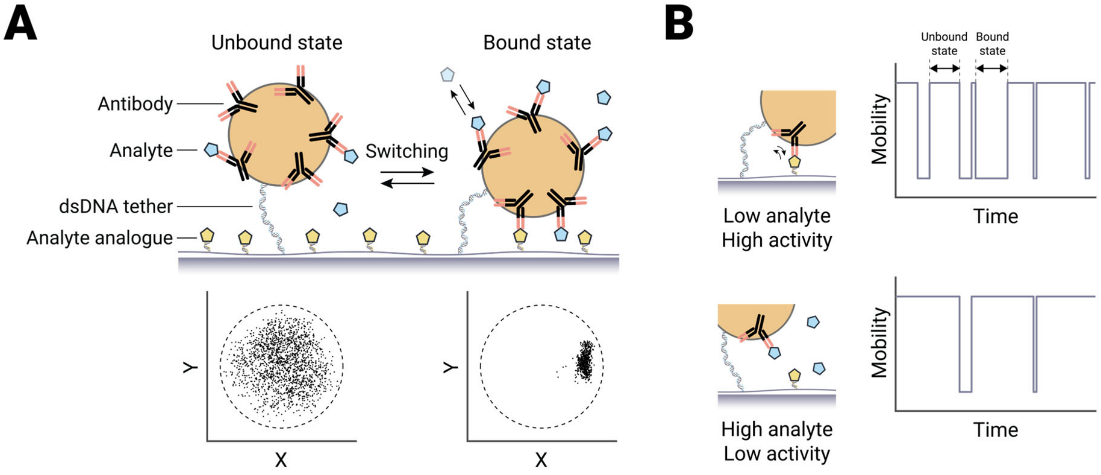


**Supplementary Figure S1 | Measurement principle of Biosensing by Particle Motion (BPM) for a competitive format.** (A) Binding states of the BPM sensor. Micrometer-sized particles (orange) are tethered to a substrate via double-stranded DNA. Particles are functionalized with antibodies that are specific to the analyte molecules in solution and analyte analogue molecules that are attached to the substrate. The transient binding of particle-side antibodies to the substrate-side analyte-analogue molecules limits the Brownian motion of the particle, as seen in the motion patterns of the particle, i.e., the projection of the center of the particle in the xy-plane. The mobility of the particle either corresponds to an unbound state (high mobility, left) or a bound state (low mobility, right). (B) Sketch of the signals of the BPM sensor. Digital switching events from the unbound to bound state and vice versa are identified by tracking the mobility of the particle over time. Low and high concentrations of analyte in solution result in a high and low frequency of switching events, respectively.

Biosensing by Particle Motion (BPM) is a biosensing technology that relies on detecting single-molecule interactions by observing the motion of micrometer-sized particles (Figure S1). In the competition format of the BPM assay (Figure S1a), the particles are provided with binder molecules (here illustrated as antibodies) and the substrate is provided with analyte-analogue molecules. The binder molecules can transiently bind to the analogue molecules, but also to analyte molecules in solution. Particles that do not interact with analogue molecules exhibit a large motion pattern, that relates to the flexibility of the double-stranded DNA tether. The mobility of the particles becomes more confined upon binding of the particle-side binders to the analogue molecules. The changes in motion are detected using widefield microscopy. Figure S1b illustrates the mobility of the particle over time at low (top) and high (bottom) concentrations of analyte. A high frequency of binding and unbinding events, i.e., a high switching activity, is observed when no analyte is present. The activity decreases with higher analyte concentrations, as the analyte blocks the binding sites of the antibodies and in that way decreases the probability per unit time that the particle can bind to the substrate.

## S2. Selected recombinant antibodies from phage display libraries after ELISA selection.

**Supplementary Table S1 | Selected recombinant antibodies from phage display libraries after ELISA selection.** The second column indicates the expression formats.

| Antibody | Format |
|---|---|
| 1 | $(VHH)_2$-Fc |
| 2 | $(VHH)_2$-Fc |
| 3 | $(VHH)_2$-Fc |
| 4 | $(VHH)_2$-Fc |
| 5 | $(VHH)_2$-Fc |
| 6 | $(VHH)_2$-Fc |
| 7 | $(scFv)_2$-Fc |
| 8 | $(scFv)_2$-Fc |
| 9 | $(VHH)_2$-Fc |
| 10 | $(VHH)_2$-Fc |
| 11 | $(VHH)_2$-Fc |
| 12 | $(scFv)_2$-Fc |
| 13 | $(VHH)_2$-Fc |

## S3. Response of selected anti-solanidine antibodies upon the addition of α-solanine using fBPM microtiter screening

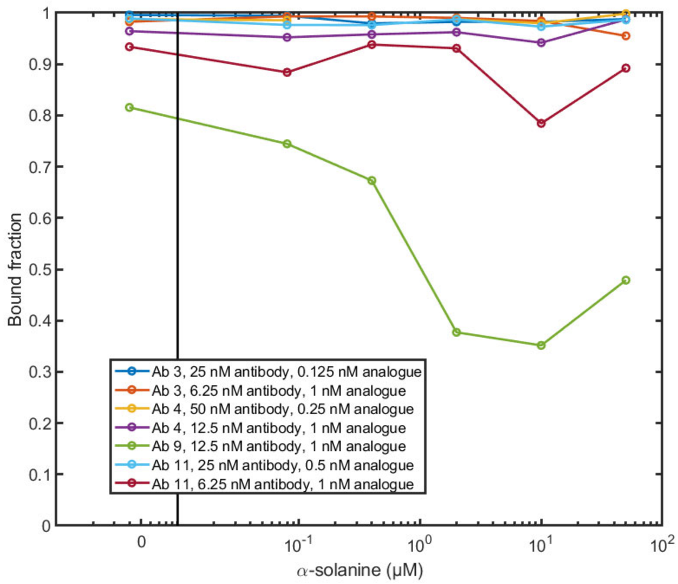


**Supplementary Figure S2 | Response of selected anti-solanidine antibodies upon the addition of α-solanine using fBPM microtiter screening.** Anti-solanidine $(scFv)_2$-Fc $(VHH)_2$-Fc antibodies were physisorbed onto the well-plate surface, after which solanidine-coated particles were added. The fraction of unbound particles was obtained through the analysis of the diffusion coefficient of each individual particle. Several antibody and analogue concentrations were varied, as indicated in the legend.

## S4. Correction of raw measurement data of α-solanine and α-chaconine

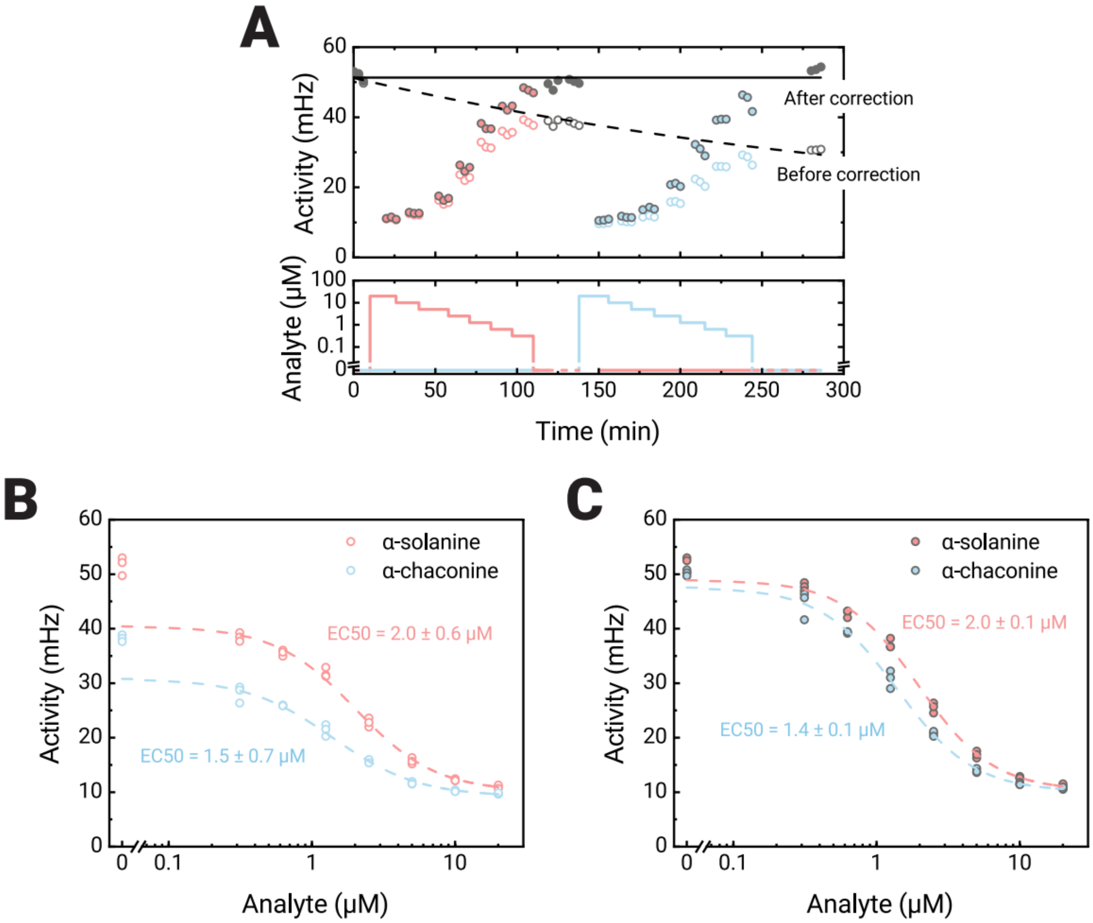


**Supplementary Figure S3 | Correction of raw measurement data of α-solanine and α-chaconine.** (A) Switching activity over time. The bottom panel shows the concentration of α-solanine (red) and α-chaconine (blue) over time, the top panel shows the raw (open circles) and corrected (filled circles) response of the sensor. It can be seen that the activity signal of the blank measurements decays over time. This may be caused by a slow loss of binder molecules[8]. The raw data $A(t)$ is corrected by fitting a single exponential decay function ($A(t) = a \cdot e^{-k_{decay}t} + A_0$) through the blank measurement data (dashed line) and applying the obtained constants to the raw data: $A_{corr}(t) = (A(t) - A_0) \cdot e^{k_{decay}t} + A_0$, with $k_{decay}$ being the fitted decay rate constant and $A_0$ the baseline activity in the absence of solanidine-analogue molecules. (B) Dose-response curve for α-solanine (red) and α-chaconine (blue), obtained with the raw data from panel a. The data points were fitted with a sigmoidal curve ($y = a + (b - a) \cdot \frac{x^n}{x^n + EC50^n}$), resulting in EC50 values of 2.0 ± 0.6 μM and 1.5 ± 0.7 μM (fitted value ± standard error of the fitted value) for α-solanine and α-chaconine, respectively. (C) Dose-response curve for α-solanine (red) and α-chaconine (blue), obtained with the corrected data from panel a. The data points were fitted with a sigmoidal curve, resulting in EC50 values of 2.0 ± 0.1 μM and 1.4 ± 0.1 μM (fitted value ± standard error of the fitted value) for α-solanine and α-chaconine, respectively.

Signal drift within BPM relates to the loss of switching activity over time, presumably related to a loss of binder molecules[8]. This can be clearly seen when comparing the switching activity of the system in the absence of analyte (Figure S3a, grey open circles) at different points in time. In order to improve the operational lifetime of the BPM sensor, we make use of the predictable nature of the signal drift to apply a mathematical correction to the raw data. Here, we fit the blank measurement data with a single exponential decay function $A(t) = a \cdot e^{-k_{decay}t} + A_0$ (Figure S3a, dashed line), with $a$ being the amplitude of the BPM signal at the start of the measurement, $k_{decay}$ the observed decay rate and $A_0$ the baseline activity in the absence of solanidine-analogue molecules. The corrected activity signal $A_{corr}$ can then be obtained by applying these constants to the raw data: $A_{corr}(t) = (A(t) - A_0) \cdot e^{k_{decay}t} + A_0$.

The result of this correction method on measurements of analyte concentrations can be seen in Figure S3b and Figure S3c, which show measured activity values of known α-solanine and α-chaconine samples using raw and corrected data, respectively. The raw data shown in Figure S3b shows an apparent difference in sensor response to α-solanine and α-chaconine. However, the corrected data shown in Figure S3c indicates that these differences are largely caused by the time-dependent signal drift.

## S5. Analysis of state lifetimes in the presence of α-solanine and α-chaconine

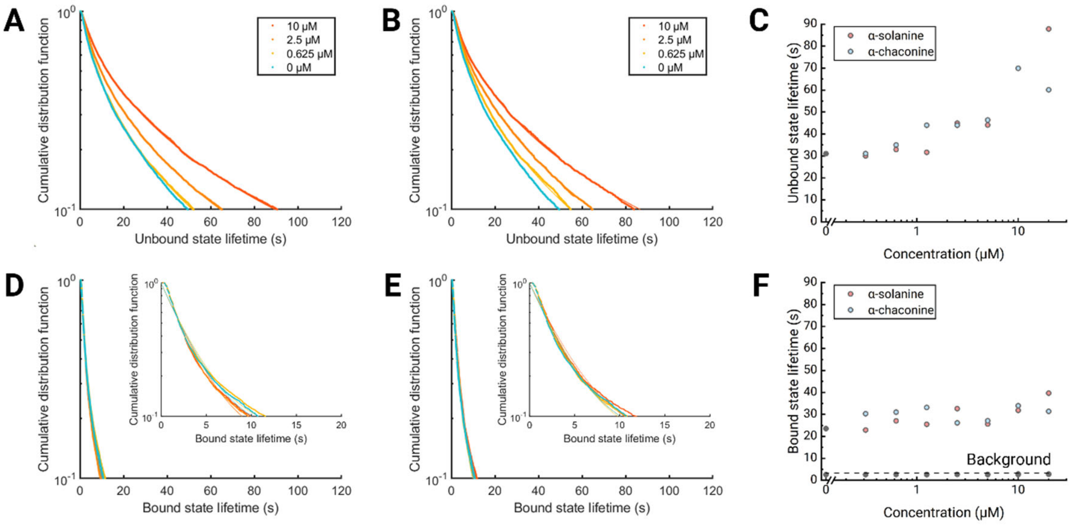


**Supplementary Figure S4 | Analysis of state lifetimes in the presence of α-solanine and α-chaconine.** (A) Unbound-state lifetime survival curves for different α-solanine concentrations. (B) Unbound-state lifetime survival curves for different α-chaconine concentrations. (C) Characteristic unbound-state lifetimes as function of the glycoalkaloid concentration. The characteristic state lifetime is obtained by fitting the unbound-state lifetime survival curves with a multiexponential fit that takes into account the heterogeneities of particle and surface binder densities [7,15]. (D) Bound-state lifetime survival curves for different α-solanine concentrations. (E) Bound-state lifetime survival curves for different α-chaconine concentrations. (F) Characteristic bound-state lifetimes as function of the glycoalkaloid concentration. The characteristic state lifetime is obtained by fitting the bound-state lifetime survival curves with a double-exponential fit that differentiates between specific interactions of the solanidine-analogue and anti-solanidine antibodies, and a background contribution caused by algorithm artefacts and nonspecific binding events (grey data points). The dashed line corresponds with the obtained characteristic bound-state lifetime of the BPM system in the absence of analogue molecules, obtained with a single-exponential fit.
